# Haematological profile of children with malaria in Sorong, West Papua, Indonesia

**DOI:** 10.1186/s12936-021-03638-w

**Published:** 2021-03-04

**Authors:** Syilvia Jiero, Ayodhia Pitaloka Pasaribu

**Affiliations:** 1 Department of Child Health, Sorong Regional General Hospital, Sorong, West Papua Indonesia; 2 Department of Child Health, Medical Faculty, Universitas Sumatera Utara, Dr. Mansur Street No. 5, 20156 Medan, Indonesia

**Keywords:** Haematological profile, *Plasmodium falciparum*, *Plasmodium vivax*, Malaria, West Papua

## Abstract

**Background:**

Malaria remains a major public health problem in Indonesian Papua, with children under five years of age being the most affected group. Haematological changes, such as cytopenia that occur during malaria infection have been suggested as potential predictors and can aid in the diagnosis of malaria. This study aimed to assess the haematological alterations associated with malaria infection in children presenting with signs and symptoms of malaria.

**Methods:**

A retrospective study was performed by collecting data from the medical records of malaria patients at Sorong Regional General Hospital, Sorong, West Papua, Indonesia, both from outpatient and inpatient clinics, from January 2014 until December 2017. The laboratory profile of children suffering from malaria was evaluated.

**Results:**

One hundred and eighty-two children aged 1 month to 18 years old were enrolled. The subjects were mostly male (112, 61.5%) with a mean age of 6.45 years (SD = 4.3 years). Children below 5 years of age suffered the most from malaria in this study (77, 42.3%). One hundred two subjects (56%) were infected with *Plasmodium falciparum*. Half of the enrolled subjects (50%) had haemoglobin level (Hb) between 5.1 and 10 gr/dL. A total of 41 children (53.2%) less than 5 years old suffered from *P. falciparum* infection. In the age group of 5–10 years, there were 34 children (57.6%) who suffered from *P. falciparum*, and in the age group > 10 years, 27 children (58.7%) suffered from *P. falciparum* infection. Only 4 subjects (5.2%) in the less than 5 years old age group had mixed malaria infection. Among eight predictors of the haematological profile, there were five predictors that were significantly associated with the diagnostic criteria, namely haemoglobin, haematocrit, leukocytes, platelets and monocytes (p < 0.05). Generally, clinical symptoms are not significantly associated with a malaria diagnosis, and only one variable showed a significant relationship, pale, with a P value of 0.001.

**Conclusions:**

Children with malaria had changes in some haematological markers, with anaemia, low platelet count, white blood count, and lymphocyte count being the most important predictors of malaria infection in the study area. These markers could be used to raise suspicion of malaria in children living in high endemic areas, such as West Papua.

## Background

Malaria is a life-threatening protozoan disease caused by parasites that are transmitted to humans through the bite of infected female *Anopheles* mosquitoes [1]. The World Health Organization (WHO) estimates that there were 229 million cases of malaria in worldwide 2019, with 409,000 deaths, most of which occurred in Africa, followed by SouthEast Asia. 67% of all malaria deaths were in children under 5 year of age. Thus, malaria continues to be viewed as a highly significant disease of global public health importance [2]. There are six major *Plasmodium* species infecting humans: *Plasmodium falciparum, Plasmodium vivax, Plasmodium malariae Plasmodium ovale curtisi*, *Plasmodium ovale wallikeri* and *Plasmodium knowlesi* [3]. *Plasmodium falciparum* and *P. vivax* are the two main causes of human malaria infections. Falciparum malaria poses a risk of severe complications and contributes to the majority of deaths [4].

In Southeast Asia, Indonesia contributes 9 % of all malaria cases and has the second highest burden of disease after India [5]. The WHO estimated that 27% of the 257,563,815 people in the Indonesian population lives in malaria endemic areas. One of the endemic areas of malaria in Indonesia is Papua [6].

**Fig. 1:**
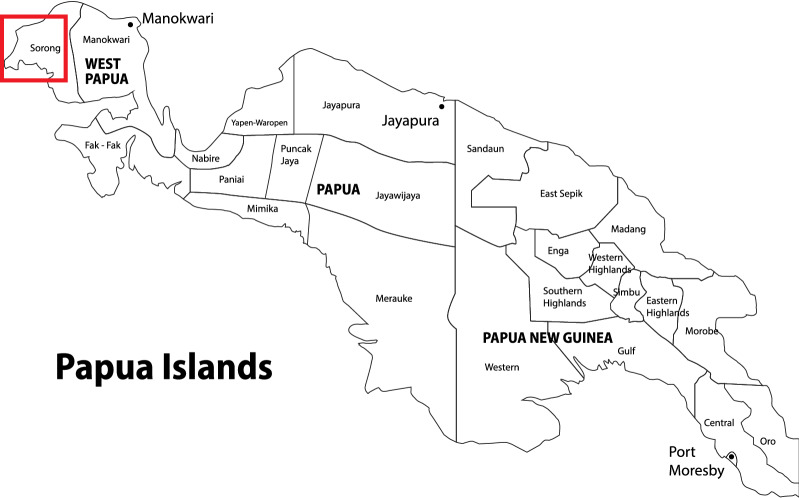
Location of study area in Sorong, West Papua, Indonesia

West Papua is an Indonesian province located on the western tip of Papua Island, which capital city is Manokwari. The area of the province is about 99671,63 km^2^ covering the bird’s head area of Papua island and the surrounding islands [7]. Geographically, Sorong city (Fig. 1) is located at the coordinates 131° 51′ East Longitude and 0° 54′ South Latitude with an area of ​​1105 km^2^ with population density about 365 persons per square kilometre consisting of 30 districts and 26 sub-districts. The population in Sorong city is about 239,815 people in 2017 with the number of villages in about 226 villages and 11 community health centres [8]. As many as 70–75% of Papuans live in rural areas. The combination of geographic features, climate conditions and extreme poverty provides a suitable environment for malaria transmission, both biologically and socially [9].

Based on the Ministry of Health of the Republic of Indonesia, Annual Parasite Index (API) for West Papua in 2017 was 15‰ and classified by WHO as hyper-endemic area. Specifically API for Sorong city was 1.5‰ and classified by WHO as meso-endemic area. The number of confirmed malaria cases microscopically in 2017 was 13,690 cases and for the city of Sorong was 127 cases [10].

Haematological alterations that are thought to characterize malaria are related to the overt biochemical changes that occur during the asexual stage of the life cycle of the malaria parasite [11]. Patients infected with malaria tend to have significantly lower platelet, leukocyte, lymphocyte, eosinophil, red blood cell, and haemoglobin (Hb) counts, while the number of monocytes and neutrophils was significantly higher than that in nonmalaria-infected patients [12–15]. Anaemia [16], leukopenia [17, 18] and thrombocytopenia [19, 20] are commonly seen in *P. falciparum* infection, probably as a result of the higher levels of parasitaemia found in these patients [21, 22]. Thrombocytopenia is most commonly seen in malaria infection [14, 23, 24]. People with platelet counts < 150,000/µL are 12–15 times more likely to develop malaria infection than people with a platelet count > 150,000/µL [14]. Pancytopenia and bicytopenia are common haematological problems encountered in clinical practice [12, 13] that have multiple causes, and the underlying pathology determines the potential predictors and can aid in the diagnosis, management and prognosis of malaria [25, 26]. It plays a major role in fatality [27, 28]. When compared across different malarial species, haematological parameters count also vary significantly [29, 30].

The prediction of haematologic changes in malaria allows clinicians to establish early and effective therapeutic interventions to prevent major complications, especially in nonendemic countries with declining malaria transmission and nonendemic countries [31]. Haematologic parameters can help provide a presumption of treatment, especially if the results of parasitological examinations are not immediately available or are uncertain to decide malaria treatment [13, 31, 32], and help intensively treat the patient and prevent possible death occurs as a result of these complications [33].

The gold standard for malaria examination is microscopic slide examination [34]. Knowledge of changes in various haematological parameters in children suffering from malaria can increase the diagnosis of malaria by increasing suspicion of malaria and encouraging a careful search for parasitaemia using a microscope [25]. There has been no research to investigate the effects of malaria on the haematological profile of Indonesian Papuan children.

## Methods

A retrospective study of data from medical records of malaria patients at Sorong Regional General Hospital, Sorong, West Papua, Indonesia Outpatient and Inpatient Clinic from January 2014 until December 2017 was performed. It included 182 children from 1 month to 18 years old with signs and symptoms of malaria at Sorong Regional General Hospital, Sorong, West Papua, Indonesia. Blood samples were collected from children into ethylenediaminetetraacetic acid (EDTA) tubes and used to prepare thin and thick blood films, which were then used for Giemsa microscopy to detect malaria parasites and malaria species. Patients’ haematological predictors were determined using an automated haematology analyzer.

### Study population

The study involved children aged 1 month to 18 years, who were malaria-confirmed by microscopy. Individual data was obtained from medical records. The study included children who were treated as inpatients or outpatients at Sorong Regional General Hospital, Sorong, West Papua, Indonesia from January 2014 until December 2017. The study was approved by the Research Ethics Committee of the Medical School of the Universitas of Sumatera Utara.

### Study design

#### Inclusion criteria

- Children in age group 1 month to 18 years.
- Any acute febrile illness lasting for 2–7 days.
- Peripheral blood smear or rapid malaria antigen test positive for *P. vivax* and/or *P. falciparum* malaria.
- Complete medical record data..

#### Exclusion criteria

- No malaria found from either rapid diagnostic test (RDT) or microscopic examination..

### Diagnosis

#### Sample collection

Two to three millilitres of venous blood were collected from each participant using a 5 ml sterile disposable syringe and dispensed into an EDTA anticoagulated test tube, followed by preparation of the thick and thin smears and automated for determination of the complete blood count (CBC). The EDTA test tubes containing the blood samples were gently inverted approximately 8 times to ensure the complete mixture of blood cells. The blood samples were collected by a trained medical laboratory scientist. Blood counts were performed using Sysmex XP 100 Haematology Analyzer.

#### Peripheral blood smear examination

Peripheral blood smears were prepared using venous blood samples. Separate slides were used for thick and thin smears. Examination of thin and thick blood films were based on WHO 2010 guidelines [35]. Thick and thin films of peripheral smear (PS) examination blood slides were prepared and stained with Giemsa. Peripheral blood smear examination for the type of malaria parasite was performed systematically under low power, high power and oil immersion using an Olympus CX21 Microscope.

### Definitions

- Definition of anaemia was based on WHO guidelines with different cut off for different agegroup and categorized by different severity [36] .
- Lymphocytopenia in chidren is defined as a total lymphocyte count less than 3.0 × 10^9^/L [37].
- Leukopenia was defined as white blood cells (WBCs) < 4 × 10^3^/µl [29].
- Monocytosis was defined as an absolute monocyte count > 3 × 10^3^/µl [37].
- Thrombocytopenia was defined as platelet count < 150 × 10^3^/µl [29].
- Categorization of malaria into uncomplicated and severe forms [38].
- Severe malaria (SM) was defined by the presence of asexual parasitaemia in addition to at least one or more of the following WHO [39, 40]. The criterias for severe malaria follows the 2010 WHO guidelines [41].
- Uncomplicated malaria (UM) was defined as the presence of symptoms and/or signs of malaria and a positive parasitologic test in the absence of evidence of end organ damage [40].

### Statistical analysis

The data are presented as the means, percentages, standard deviations, medians, and ranges. Statistical analysis was performed using ANOVA, the Kruskal-Wallis test, contingency coefficient correlation, the Mann-Whitney U test, and the independent t-test. The data were analysed using SPSS version 21 statistical software with appropriate statistical methods. Differences with P-values of less than 0.05 were considered significant.

## Results

### Characteristics and haematological profile of research subjects

This study included 182 subjects who met the inclusion and exclusion criteria. The subjects were mostly male (112, 61.5%) with a mean age of 6.45 years (SD = 4.3 years). Children < 5 years accounted for the majority of the cases, (77, 42.3%). A total of 102 subjects (56%) were infected with *P. falciparum*. Some subjects (50%) had a Hb level between 5.1 and 10 gr/dL. The results of the haematological profile examination of the subjects are presented in Table 1 below.

**Table 1:** Characteristics and hematological profile of subjects with malaria

Characteristics	n = 182
Sex, n (%)	
Male	112 (61.5)
Female	70 (38.5)
Age, mean (SD), years	6.45 (4.30)
Age group	
> 10 years	46 (25.3)
5–10 years	59 (32.4)
< 5 years	77 (42.3)
Weight, mean (SD), kg	18.95 (11.27)
Type of malaria	
*Plasmodium vivax*	76 (41.8)
*Plasmodium falciparum*	102 (56)
Mixed malaria	4 (2.2)
Hemoglobin, n (%)	
> 10 gr/dL	77 (42.3)
5.1–10 gr/dL	91 (50)
≤ 5 gr/dL	14 (7.7)
Leukocytes, n (%)	
> 11,000	35 (19.2)
6000–11,000	73 (40.1)
< 6000	74 (40.7)
Platelets, n (%)	
> 100.000	126 (69.2)
≤ 100.000	56 (30.8)
Anemia, n (%)	
Yes	140 (76.9)
No	42 (23.1)
Anemia grade, n (%)	
No anemia	42 (23.1)
Mild	27 (14.8)
Moderate	68 (37.4)
Severe	45 (24.7)
Leucopenia, n (%)	
Yes	31 (17)
No	151 (83)
Monocytosis, n (%)	
Yes	53 (29.1)
No	129 (70.9)
Lymphocytopenia, n (%)	
Yes	120 (65.9)
No	62 (34.1)
Thrombocytopenia, n (%)	
Yes	97 (53.3)
No	85 (46.7)
Hepatomegaly, n (%)	
Yes	41 (22.5)
No	141 (77.5)
Splenomegaly, n (%)	
Yes	34 (18.7)
No	148 (81.3)

Forty-one subjects who were less than five years old suffered from *P. falciparum* infection (53.2%). Only four subjects (5.2%) less than five years old had mixed infections (Table 2). Table 3 shows the haematological profiles based on the age group of the study subjects. Of the eight predictors in the haematological profile, there are five predictors that were significantly associated with the diagnostic criteria, namely haemoglobin, haematocrit, leukocytes, platelets and monocytes (p < 0.05) (Tables 4). Anemia (p < 0.001) and thrombocytopenia (p = 0.011) were significantly more common in *P. falciparum* as compared to *P. vivax* infection in uncomplicated malaria (Table 5).

**Table 2:** Results of malaria diagnosis by age group

Age group, n (%)	*P. vivax*	*P. falciparum*	Mixed malaria	p-value*
< 5 years	32 (41.6)	41 (53.2)	4 (5.2)	0.226
5–10 years	25 (42.4)	34 (57.6)	0 (0)	
> 10 years	19 (41.3)	27 (58.7)	0 (0)	

*Contingency coefficient correlation

**Table 3:** Hematology profile based on age group

Hematology profile	< 5 years	5–10 years	> 10 years
Hemoglobin (g/dL), mean (SD)	8.71 (2.92)	9.51 (2.62)	9.73 (2.09)
Hematocrit (%), mean (SD)	27.81 (9.10)	30.38 (8.42)	30.99 (6.99)
Erythrocytes (x 10^6^/µL), median (range)	4.17 (1.33–8.05)	4.27 (1.75–7.11)	4.16 (2.55–5.69)
Leukocytes (x 10^3^/µL), median (range)	8.1 (1.9-29.17)	5.89 (2-20.4)	5.50 (2-17.9)
Platelets (x 10^3^/µL), median (range)	168 (29–718)	154 (13–391)	105 (41–470)
Neutrophil (x 10^3^/µL), median (range)	51.1 (23.2–89.9)	48.7 (26–84,5)	54.15 (25.9–88)
Lymphocytes (x 10^3^/µL), median (range)	38.8 (4.7–77.9)	41.2 (12.3–88.5)	35.7 (9.8–82.4)
Monocytes (x 10^3^/µL), median (range)	7.5 (2-64.3)	7.8 (2.8–27.2)	8.35 (2.9–28.5)

**Table 4:** Comparison of hematology profiles with different malaria species

Hematology profile	*P. vivax*	*P. falciparum*	Mixed malaria	p-value
Hemoglobin (g/dL), median (IQR)	10.25 (8.73–11.48)	8.70 (6.80–11.05)	6.05 (3.25–12.15)	0.018^a^
Hematocrit (%), median (IQR)	31.31 (8.066)	28.33 (8.27)	22.45 (13.99)	0.016^b^
Erythrocytes (x 10^6^/µL), median (IQR)	4.34 (3.99–4.76)	3.89 (3.19–4.76)	3.42 (1.81–5.49)	0.059^a^
Leukocytes (x 10^3^/µL), median (IQR)	7.2 (5.1–10.150)	5.55 (3.953–8.43)	11.4 (6.28–14.95)	0.006^a^
Platelets (x 10^3^/µL), median (IQR)	189 (106–236.5)	118.5 (817.5–175.75)	186.5 (74.75–226.25)	0.01^a^
Neutrophil (x 10^3^/µL), median (IQR)	53.15 (39.53–66.70)	49.65 (38.35–67.43)	50.95 (44.00–61.88)	0.942^a^
Lymphocytes (x 10^3^/µL), median (IQR)	38.35 (27.05–52.00)	40.40 (24.28–50.80)	41.25 (30.45–46.28)	0.910^a^
Monocytes (x 10^3^/µL), median (IQR)	6.60 (4.55–11.83)	8.60 (6.15–12.65)	7.80 (3.18–9.73)	0.031^a^

^a^Kruskal-Wallis, ^b^ANOVA

**Table 5:** Comparison of characteristics with different species of malaria

Characteristics of subjects	*P. vivax*	*P. falciparum*	Mixed malaria	p-value
Sex, n (%)				
Male	49 (26.9)	60 (33)	3 (1.6)	0.637^a^
Female	27 (14.8)	42 (23.1)	1 (0.5)	
Age (years), median (range)	6.67 (0.33-14)	6 (0.08–15.67)	1.88 (1.42–3)	0.059^b^
Age group, n (%)				
< 5 years	32 (17.6)	41 (22.5)	4 (2.2)	0.226^a^
5–10 years	25 (13.7)	34 (18.7)	0 (0.0)	
> 10 years	19 (10.4)	27 (14.8)	0 (0.0)	
Weight (kg), mean (SD)	18.71 (11.77)	19.49 (10.99)	9.7 (0.36)	0.069^b^
Hemoglobin, n (%)				
≤ 5 g/dL	3 (1.6)	9 (4.9)	2 (1.1)	0.005^a^
5.1–10 g/dL	34 (18.7)	56 (30.8)	1 (0.5)	
> 10 g/dL	39 (21.4)	37 (20.3)	1 (0.5)	
Leukocytes, n (%)				
< 6000 g/dL	23 (12.6)	50 (27.5)	1 (0.5)	0.065^a^
6000–11.000 g/dL	36 (19.8)	36 (19.8)	1 (0.5)	
> 11.000 g/dL	17 (9.3)	16 (8.8)	2 (1.1)	
Platelets, n (%)				
≤ 100.000 g/dL	18 (9.9)	37 (20.3)	1 (0.5)	0.192^a^
> 100.000 g/dL	58 (31.9)	65 (35.7)	3 (1.6)	
Anemia, n (%)				
Yes	54 (29.7)	83 (45.6)	3 (1.6)	0.270^a^
No	22 (12.1)	19 (10.4)	1 (0.5)	
Thrombocytopenia, n (%)				
Yes	31 (17)	65 (35.7)	1 (0.5)	0.005^a^
No	45 (24.7)	37 (20.3)	3 (1.6)	
Grading anemia, n (%)				
No anemia	22 (12.1)	19 (10.4)	1 (0.5)	0.256^a^
Mild	12 (6.6)	15 (8.2)	0 (0)	
Moderate	30 (16.5)	37 (20.3)	1 (0.5)	
Severe	12 (6.6)	31 (17.0)	2 (1.1)	
Severe malaria, n (%)				
Yes	9 (4.9)	13 (7.1)	2 (1.1)	0.087^a^
No	67 (36.8)	89 (48.9)	2 (1.1)	
Hepatomegaly, n (%)				
Yes	9 (4.9)	31 (17.0)	1 (0.5)	0.021^a^
No	67 (36.8)	71 (39.0)	3 (1.6)	
Splenomegaly, n (%)				
Yes	7 (3.8)	26 (14.3)	1 (0.5)	0.014^a^
No	69 (37.9)	76 (41.8)	3 (1.6)	

^a^ Contingency coefficient correlation

^b^ Kruskal-Wallis

## Discussion

Malaria is a preventable and treatable condition and remains the most important parasitic disease globally [6]. In 2017, it was still endemic in 80 countries, placing 3.7 billion people at risk. Considerable progress has been made due to aggressive malaria control and elimination efforts since 2000, resulting in a global reduction of 40% in morbidity and 60% in mortality [42]. Many efforts have been made to minimize malaria transmission worldwide; however, this infection remains high among humans [43]. All species of *Plasmodium* have been documented in Indonesia. *Plasmodium vivax* is the predominant species except in Papua where *P. falciparum* slightly predominates [5, 44, 45].

*Plasmodium falciparum* is the most prevalent malaria parasite in the WHO African region, accounting for 99.7% of estimated malaria cases in 2017, as well as in the WHO regions of South-East Asia Region (62.8%), the Eastern Mediterranean (69%) and the Western Pacific (71.9%). *Plasmodium vivax* is the predominant parasite in the WHO region of the Americas (74.1%) [42]. *Plasmodium vivax* contributed 4% of the total global cases in 2015, but outside Africa the proportion was 41% among all malaria infections. Its high burden of disease is maintained in part due to dormant liver stage parasite forms known as hypnozoites which can induce clinical relapse episodes [46].

In the present study, there were more male patients infected with malaria than female patients, which is similar to the results of a study in South Sorong [47]. One hundred two subjects (56%) were infected with *P. falciparum*. This study also shows that the most common type of *Plasmodium* infection in Sorong, West Papua, is *P. falciparum*. This is similar with other provinces in Indonesia and in Manokwari, West Papua, *P. falciparum* is the predominant malaria species [45, 48].

About 42.3% of malaria cases in our study was among children < 5 year old. According to WHO data in 2017, they are the most vulnerable group affected by malaria. They accounted for 61% (266,000) of all malaria deaths worldwide [42].

Cytopenia is a disorder in which the production of one or more blood cell types ceases or is greatly reduced. The types of cytopenia are anaemia, which is a reduction in red blood cells (RBCs); leukopenia, which is a reduction in WBCs; neutropenia (neutrophils make up over half of all WBCs), which is a deficiency in neutrophils; and thrombocytopenia, which is deficiency in platelets. Bicytopenia is defined as a condition in which two out of three cell lines (RBCs, WBCs, and platelets) are reduced. The simultaneous reduction in all three formed cell lines is termed pancytopenia [49]. These kinds of cytopenias are not uncommon in malaria; bone marrow diagnosis of adults with bicytopenia and pancytopenia has shown that 3% of bicytopenia and 6% of pancytopenia were caused by malaria [50, 51].

Anaemia is also a common manifestation, particularly in infants with *P. vivax* and in children with *P. knowlesi* infection [52–54]. Anaemia is one of the most common complications in malaria infection, especially in younger children and pregnant women in high transmission areas [55, 56].

The pathogenesis of anaemia during malaria infection is not clearly understood. However, it is estimated that the main targets of parasites are RBCs, which results in damage to RBCs, acceleration of parasite growth and nonparasitic removal [57], bone marrow dysfunction [58], and the level of parasitaemia [59]. Anaemia in malaria, however, is associated with a combination of haemolysis of parasitized RBCs, accelerated removal of both parasitized and unparasitized RBCs, depressed and ineffective erythropoiesis due to tumor necrosis factor alpha, anaemia of chronic disease, and splenic phagocytosis or pooling [60–62].

This study showed that haematological abnormalities in children with malaria infection are common. Some subjects (50%) had a Hb level between 5.1 and 10 gr/dL. The prevalence of anaemia in This study was 77.4%; 14.8% of subjects had mild anaemia, 35.7% had moderate anaemia, and 26.9% had severe anaemia. The rate of anaemia in children < 5 years old was higher than that in children 5–10 years old and > 10 years old, with a mean Hb of 8.71 gr/dL. Data from household surveys conducted in 25 high-burden African countries between 2015 and 2019 show that, among children < 5 years who tested positive for malaria, the prevalence of moderate to severe anaemia was between 9–76.3% [2]. A laboratory trial study of 30 patients in Iran observed significantly lower values of Hb/dL, haematocrit (Ht)%, mean corpuscular volume (MCV)/ fl. and mean corpuscular haemoglobin (MCH)/ µl, WBC/ µl, and platelet/ µl among malaria-infected children compared to healthy children [13, 15, 63, 64]. Our findings were consistent with these previous reports.

There are various hypotheses about thrombocytopenia that occurs in malaria infections. Thrombocytopenia seems to occur through peripheral damage [65], excessive removal of platelets by spleen pooling [66, 67] and platelet consumption by the process of disseminated intravascular coagulopathy (DIC) [68]. Sufficient or increased numbers of megakaryocytes in the bone marrow affect the decrease in thrombopoiesis, which is a possible cause of thrombocytopenia in malaria [66]. The destruction of circulating platelets mediated by immunity has been postulated as the cause of thrombocytopenia seen in malaria infections. Platelets have also been shown to mediate clumping of erythrocytes infected with *P. falciparum* [69]. This can cause apparent thrombocytopenia. Patients infected with malaria experience increased levels of specific immunoglobulin G (IgG) in the blood that binds to malaria antigens bound to platelets, which may lead to acceleration of platelet destruction [23]. Previous studies revealed that platelet aggregation, which is the clumping of platelets, was incorrectly calculated as a single platelet by the analyzer, causing pseudothrombocytopenia [13]. In addition, during malaria infection, endothelial activation is activated and can contribute to the loss of endothelium barrier function and organ dysfunction. This process can use released platelets and proteins as important regulators of endothelial permeability, resulting in thrombocytopenia [70]. However, thrombocytopenia in malaria infection has also been associated with sequestration and pooling of platelets in the spleen, immune-mediated destruction of circulating platelets, and platelets mediating the clumping of *P. falciparum-*infected erythrocytes, leading to pseudothrombocytopenia [13, 63, 69].

Thrombocytopenia was seen in 97 children (53.3%) and was highly significant in the age group > 10 years, with a median 105 × 10^3^/µL in this study.

It is found mostly in *P. falciparum* infection, as many as 65 (35.7%). Thrombocytopenia was observed in malaria-infected children in this study, which is consistent with earlier reports [13, 63]. There were both qualitative and quantitative changes in platelet abnormalities in malaria. Platelet counts were significantly reduced in children infected with malaria. A study conducted in Thailand–Myanmar border showed that thrombocytopenia occured. This observation may imply that thrombocytopenia can be a marker of *Plasmodium* infection [13, 71–73].

In the present study overall, when compared between malaria species in uncomplicated cases, Hb and platelet value in the *P. falciparum* infection was lower than *P. vivax* infection as well as the platelet value (Table 6) with p value 0.001. These figures were in good agreement with studies done by Latif et *al.* showing *P. falciparum-*associated anaemia and thrombocytopenia compared to *P. vivax* infection, (8.1 ± 2.2 vs. 11 ± 3.2 g/dL and 60.338 ± 50.6 × 10^3^/µL vs. 77.907 ± 78.4 × 10^3^/µL, respectively) [21]. A study by Hyder et al. also found the same thing with the following results 10.1921 ± 0.33157 g/dL vs. 11.3306 ± 0.29730 g/dL and 88.0239 ± 1.17476/µL x 10^3^/µL vs. 98.1763 ± 0.99502 × 10^3^/µL [30].

**Table 6:** Comparison of hematological profile between different malaria species

Hematology profile	Uncomplicated Malaria			p-value*	Complicated Malaria			p-value*
	*P. vivax*	*P. falciparum*	Mixed malaria		*P. vivax*	*P. falciparum*	Mixed malaria	
Hemoglobin (g/dL), median (IQR)	10.30 (8.80–11.40)	8.90 (6.85–11.15)	3.30 (3.20)	0.001	8.70 (4.15–12.10)	8.20 (6.20–9.95)	11.0 (8.70)	0.340
Hematocrit (%), median (IQR)	32.0 (28.0–38.0)	28.40 (22.05–35.0)	11.30 (11.0)	0.001	25.0 (12.55–33.90)	26.50 (21.80–33.20)	33.60 (26.90)	0.322
Erythrocytes (x 10^6^/µL), median (IQR)	4.38 (4.06–4.77)	3.89 (3.18–4.74)	1.88 (1.75)	0.001	3.77 (1.87–4.51)	3.90 (3.14–4.89)	5.27 (4.84)	0.124
Leukocytes (x 10^3^/µL), median (IQR)	7.30 (4.90–10.20)	5.50 (4.05–8.15)	11.40 (8.0)	0.007	7.10 (5.95–11.0)	6.70 (3.30–16.95)	10.35 (5.70)	0.904
Platelets (x 10^3^/µL), median (IQR)	189.0 (106.0–233.0)	120.0 (89.50–175.50)	204.0 (173.0)	0.011	218.0 (78.0–354.0)	109.0 (68.50–197.0)	121.0 (42.0)	0.352
Neutrophil (x 10^3^/µL), median (IQR)	51.60 (39.60–64.90)	50.40 (38.70–67.25)	44.80 (43.20)	0.810	58.90 (34.60–68.50)	48.90 (28.80–69.70)	59.75 (55.50)	0.555
Lymphocytes (x 10^3^/µL), median (IQR)	38.70 (28.70–52.0)	40.50 (24.40–48.95)	45.70 (44.70)	0.568	34.10 (25.65–52.10)	37.80 (23.15–67.80)	32.90 (28.0)	0.785
Monocytes (x 10^3^/µL), median (IQR)	6.60 (4.50–12.30)	8.50 (6.25–11.90)	9.45 (8.90)	0.092	7.20 (4.90–8.20)	11.60 (5.25–20.60)	4.35 (2.0)	0.108

*Kruskal-Wallis

On the contrary, studies, such as Goyal et al. [74], Nadwani et al. [75], Sajjanar et al. [76], George and Alexander [77], Rodriguez-Monrale et al. [78] have shown *P. vivax*-associated anaemia and thrombocytopenia. Ullah et al. showed something different where *P. falciparum-*associated anaemia (9.9 ± 4.48 g/dL vs. 10.7 ± 2.36 g/dL) and *P. vivax*-associated thrombocytopenia (135.8 ± 89.4 × 10^3^/µL vs. 222 ± 118.7 × 10^3^/µL) [29].

In the recent years, there have been reported many severe vivax malaria cases [75, 76, 79]. The current dogma has been that *P. falciparum* can be severe and life-threatening, whereas *P. vivax* tends to be mild [80]. However, there is currently growing evidence that *P. vivax* can cause significant morbidity and even mortality in endemic areas [81]. Malaria severity is a function of parasite virulence, degree of parasitaemia, sequestration and host immune competence which in turn depends on factors, namely. age, regional transmission intensity, nutritional status and genetic susceptibility [82–86]. Another hypothesis of Price et al. that an increase in the *P. vivax* parasite resistant to chloroquine in recent years is one of the main triggers of the new severe vivax malaria [82]. All these variables make the risk and spectrum of malaria complications different around the world [83–85].

Price et al. found that *P. vivax* infection associated with severe and fatal malaria particularly in young children in Timika, Papua [82]. Otherwise, severe malaria was found more in falciparum malaria cases (7.1%) than in vivax malaria (4.9%) and mixed malaria (1.1%). This study still has shortcomings where there are no data on the number of parasites and chloroquine resistance.

This study has shown, however, that there was no significant difference in total white blood cell count in malaria-infected children. Leukopenia was seen in only 17% of children. A study by Maina et al. also showed that there was no significant difference in the total white blood cell count in malaria-infected children compared to control subjects [13]. The difference in values can be related to environmental factors, socioeconomic status, or malaria immunity, among other factors [14, 57, 86]. Different views have been expressed on the total WBCs in subjects infected with malaria because leukopenia has been reported by several authors [14, 63, 73], and leukocytosis has also been documented by other authors [13, 15, 65].

Leukocyte value in *P. falciparum* (5.55 × 10^3^/µL) was lower than in *P. vivax* infection (7.2 × 10^3^/µL). Similar outcome was also reported in a study conducted by Latif et al. showing leukocyte ​​in *P. falciparum* (8,906 ± 1.06 × 10^3^/µL) compared to *P. vivax* infection (11,868 ± 2.34 × 10^3^/µL) was lower [21]. This was in contrast to the findings reported by Ullah et al.., which showed that leukocyte ​​in *P. vivax* (8.7 ± 4.5 × 10^3^/µL) was lower than in *P. falciparum* infection (10.4 ± 11.4 × 10^3^/µL) [29].

This study showed lymphocytopenia in 97 children (53.3%). The study has further revealed that there were no statistically significant differences in granulocyte and lymphocyte counts between malaria-infected and noninfected children, and these findings are in agreement with many earlier reports [13–15, 57, 72, 86–88] but disagree with the findings of George and Ewelike-Ezeani [63]. In some cases of acute malaria, however, lymphocytopenia has been reported, but this has been associated with redistribution of lymphocytes with sequestration in the spleen [89, 90].

Generally, clinical symptoms are not significantly associated with a malaria diagnosis, and only one variable shows a significant relationship, pale, with a p value of 0.001 (Table 7). The first symptoms of malaria are nonspecific and characterized by headache, fatigue, abdominal discomfort, and muscle and joint aches, followed by fever, chills, perspiration, anorexia, vomiting and worsening malaise. These features often lead to overdiagnosis of malaria in developing countries, where diagnosis is frequently based only on clinical judgment with limited resources for parasitological testing [91].

**Table 7:** Comparison of malaria symptoms and species of parasites

Symptoms	*P. vivax*	*P. falciparum*	Mixed malaria	p-value*
Fever, n (%)	75 (98.7)	102 (100.0)	4 (100.0)	0.469
Anorexia, n (%)	51 (67.1)	73 (71.6)	3 (75.0)	0.793
Asthenia, n (%)	48 (63.2)	72 (70.6)	3 (75.0)	0.549
Myalgia/ arthralgia, n (%)	47 (61.8)	72 (70.6)	3 (75)	0.444
Nausea, n (%)	50 (65.8)	60 (58.8)	1 (25.0)	0.211
Pale, n (%)	24 (31.6)	59 (57.8)	3 (75.0)	0.001
Abdominal pain, n (%)	25 (32.9)	42 (41.2)	1 (25)	0.462
Headache, n (%)	28 (36.8)	39 (38.2)	0 (0.0)	0.298
Chills, n (%)	15 (19.7)	20 (19.6)	0 (0.0)	0.614
Sweating, n (%)	8 (10.5)	11 (10.8)	0 (0.0)	0.787
Jaundice, n (%)	0 (0.0)	1 (1.0)	0 (0.0)	0.674

*Contingency coefficient correlation

Conversely, in children, these manifestations of uncomplicated malaria can be misinterpreted and attributed to other prevalent infections, such as pneumonia, gastroenteritis, and sepsis[92]. In high transmission areas, high and repeated exposure to parasites has an impact on the acquisition of immunity, resulting in a high proportion of asymptomatic infections, particularly in older children and adults [93].

There are several limitations in this study. First, this was a retrospective study. Some data of the haematological and microscopic results retrieved from medical records were not provided. Second, there was no control group in this study, therefore association between haematological markers in malaria and non-malaria infected groups could not be stated.

## Conclusions

Children infected with malaria revealed changes in some haematological markers, with anaemia, low platelet counts, white blood counts, and lymphocyte counts being the most important predictors of malaria infection in our study area. Children living in malaria high endemic areas such as West Papua who appear with acute febrile illness and either thrombocytopenia alone or in combination with anaemia should be suspected for malaria, therefore, RDT or microscopic examination must be performed. These haematological markers could raise the suspicion of malaria in this area.

## Data Availability

The datasets analysed in this study are available from the corresponding author on request.

## Abbreviations

Hb: Haemoglobin
WHO: World Health Organization
API: Annual parasite index
EDTA: Ethylenediaminetetraacetic acid
CBC: Complete blood count
PS: Peripheral smear
WBCs: White blood cells
UM: Uncomplicated malaria
Ht: Haematocrit
MCV: Mean corpuscular volume
MCH: Mean corpuscular haemoglobin
RBCs: Red blood cells
DIC: Disseminated intravascular coagulopathy
SM: Severe malaria
RDT: Rapid diagnostic test

## References

[1] WHO. World malaria report 2015. Geneva: World Health Organization; 2015. https://apps.who.int/iris/bitstream/handle/10665/200018/9789241565158_eng.pdf;jsessionid=A63034A5EF79C858F9DE80D3349C82AD?sequence=1. Accessed 15 July 2020.

[2] WHO. World malaria report 2020. Geneva: World Health Organization; 2020. https://www.who.int/docs/default-source/malaria/world-malaria-reports/9789240015791-double-page-view.pdf?sfvrsn=2c24349d_5. Accessed 6 January 2021.

[3] Sutherland CJ, Tanomsing N, Nolder D, Oguike M, Jennison C, Pukrittayakamee S (2010). Two nonrecombining sympatric forms of the human malaria parasite *Plasmodium ovale* occur globally. J Infect Dis.

[4] White NJ, Pukrittayakarnee S, Hien TT, Faiz MA, Makuolu OA, Dondorp AM. Malaria Lancet. 2013;383:723–35. 10.1016/S0140-6736(13)60024-0 23953767

[5] WHO. National malaria control programme review: Republic of Indonesia. Indonesia. WHO Country Office for Indonesia; 2011. https://apps.who.int/iris/bitstream/handle/10665/253960/9789791947749-eng.pdf;jsessionid=DABF97E279DB585057E086467F1EAB5A?sequence=1. Accessed 20 July 2020.

[6] WHO. World malaria report 2016. Geneva: World Health Organization; 2016. https://apps.who.int/iris/bitstream/handle/10665/252038/9789241511711-eng.pdf?sequence=1. Accessed 15 July 2020.

[7] Supriyanto D, Bachtia A. Achievement of malaria control program in West Papua 2012–2016. Indonesia: The 5th International Conference on Public Health; 2019. 10.26911/theicph.2019.01.25. Accessed 6 January 2021.

[8] BPS. Badan Pusat Statistik Kota Sorong. 2020. https://sorongkota.bps.go.id/. Accessed 6 January 2021.

[9] Hanandita W, Tampubolon G (2016). Geography and social distribution of malaria in Indonesia Papua: a cross-sectional study. Int J Health Geogr.

[10] Ministry of Health of the Republic of Indonesia. West Papua provincial government health profile health office 2018. Indonesia; 2018 (government document, unpublished).

[11] Muwonge H, Kikomeko S, Sembajjwe LF, Seguya A, Namugwanya C (2013). How reliable are hematological parameters in predicting uncomplicated *Plasmodium falciparum* malaria in an endemic region?. ISRN Trop Med.

[12] Bakhubaira S (2013). Hematological parameters in severe complicated *Plasmodium falciparum* malaria among adults in Aden. Turk J Haematol.

[13] Maina RN, Walsh D, Gaddy C, Hongo G, Waitumbi J, Otieno L (2010). Impact of *Plasmodium falciparum* infection on haematological parameters in children living in Western Kenya. Malar J.

[14] Erhart LM, Yingyuen K, Chuanak N, Buathong N, Laoboonchai A, Miller RS (2004). Hematologic and clinical indices of malaria in a semi-immune population of western Thailand. Am J Trop Med Hyg.

[15] Adedapo AD, Falade CO, Kotila RT, Ademowo GO (2007). Age as a risk factor for thrombocytopenia and anaemia in children treated for acute uncomplicated falciparum malaria. J Vector Borne Dis.

[16] Helegbe GK, Goka BQ, Kurthals JA, Addae MM, Ollaga E, Tetthe JK (2007). Complement activation in Ghanaian children with severe *Plasmodium falciparum* malaria. Malar J.

[17] McKenzie FE, Prudhomme WA, Magill AJ, Forney JR, Permpanich B, Lucas C (2005). White blood cell counts and malaria. J Infect Dis.

[18] Alves-Junior ER, Gomes LT, Ribatski-Silva D, Mendes CRJ, Leal-Santos FA, Simões LR (2014). Assumed white blood cell count of 8,000 cells/µL overestimates malaria parasite density in the Brazilian Amazon. PLoS ONE.

[19] Lacerda MV, Mourᾶo MP, Coelho HC, Santos JB (2011). Thrombocytopenia in malaria: who cares?. Mem Inst Oswaldo Cruz.

[20] Horstmann RD, Dietrich M, Bienzle U, Rasche H (1981). Malaria-induced thrombocytopenia. Ann Hematol.

[21] Latif I, Jamal A (2015). Hematological changes in complete blood picture in paedriatric patients of malaria caused by *Plasmodium vivax* and *falciparum*. J Ayub Med Coll Abbottabad.

[22] Chianura L, Errante IC, Travi G, Rossotti R, Puoti M (2012). Hyperglycemia in severe falciparum malaria: a case report. Case Rep Crit Care.

[23] Moulin F, Lesage F, Legros AH, Maroga C, Moussavou A, Guyon P (2003). Thrombocytopenia and *Plasmodium falciparum* malaria in children with different exposures. Arch Dis Child.

[24] Mahmood A, Yasir M (2008). Thrombocytopenia: a predictor of malaria among febrile patient in Liberia. Infect Dis J Pakistan.

[25] Njunda AL, Ngouadjeu DTE, Nsagha DS, Nyanjoh EM, Kwenti TD, Assob NJC (2016). Haematological profile of children with malaria in Kumba Health District, South West Region Cameroon. Afr J Integ Health.

[26] Kumar R, Kalra SP, Kumar H, Anand AC, Madan H (2001). Pancytopenia–a six year study. J Assoc Phys India.

[27] Zaki SA, Shanbag P (2011). Atypical manifestations of malaria. Res Rep Trop Med.

[28] Kaewpitoon S, Rujiragul R, Namwichaisirikul N, Churproong S, Ueng-arporn N, Matrakool L (2012). Malaria in Thailand 2007–2011. Srinagarind Med J.

[29] Ullah I, Ali MU, Ali S, Rafiq A, Sattar Z, Hussain S (2018). Hematological profile of patients having malaria-positive peripheral blood smears: a cross-sectional study at a diagnostic research center in Khyber Pakhtunkhwa, Pakistan. Cureus.

[30] Hyder A, Mahmood A, Mahmood R, Iqbal MI (2020). Evaluation of haematological parameters in malaria infection and its association with different species of malarial parasite. Pak Armed Forces Med J.

[31] D’acremont V, Landry P, Mueller I, Pécoud A, Genton B (2002). Clinical and laboratory predictors of imported malaria in an outpatient setting: an aid to medical decision making in returning travelers with fever. Am J Trop Med Hyg.

[32] Murphy GS, Oldfield EC (1996). Falciparum malaria. Infect Dis Clin North Am.

[33] Bidaki Z, Dalimi A (2003). Biochemical and hematological alteration in vivax malaria in Kahnouj city. J Rafsanjan Univ Med Sci.

[34] WHO. World malaria report 2010. Geneva: World Health Organization; 2010. https://www.who.int/malaria/world_malaria_report_2010/worldmalariareport2010.pdf. Accessed 15 July 2020.

[35] WHO. Methods manual microscopy for the detection, identification and quantification of malaria parasites on stained thick and thin blood films in research settings procedure. Geneva: Research Malaria Microscopy Standards Working Group; 2015. https://apps.who.int/iris/bitstream/handle/10665/163782/9789241549219_eng.pdf;jsessionid=070C9A2E74C3C475EEFC56E301155262?sequence=1. Accessed 6 January 2021.

[36] WHO. Haemoglobin concentrations for the diagnosis of anaemia and assessment of severity. Geneva, World Health Organization; 2011. https://apps.who.int/iris/handle/10665/85839. Accessed 6 January 2021.

[37] Tobón-Castaño A, Mesa-Echeverry E, Miranda-Arboleda AF (2015). Leukogram profile and clinical status in vivax and falciparum malaria patients from Colombia. J Trop Med.

[38] Kwenti TE, Kwenti TDB, Latz A, Njunda LA, Nkuo-Akenji T (2017). Epidemiological and clinical profile of paediatric malaria: a cross sectional study performed on febrile children in five epidemiological strata of malaria in Cameroon. BMC Infect Dis.

[39] WHO (2000). Severe falciparum malaria. World Health Organization, Communicable diseases cluster. Trans R Soc Trop Med Hyg.

[40] Cohee LM, Laufer MK (2017). Malaria in children. Pediatr Clin North Am.

[41] WHO. Management of severe malaria—a practical handbook, 3rd edn. Geneva: World Health Organization; 2012. https://www.who.int/malaria/publications/atoz/9789241548526/en/. Accessed 6 January 2021.

[42] WHO. World malaria report 2018. Geneva: World Health Organization; 2018. https://apps.who.int/iris/bitstream/handle/10665/275867/9789241565653-eng.pdf. Accessed 15 July 2020.

[43] Bawah AT, Nyakpo KT, Ussher FA, Alidu H, Dzogbo JJ, Agbemenya S, Kwasie DA, Seini MM (2018). Hematological profile of children under five years with malaria at the Ho Municipality of Ghana. Edorium. J Pediatr..

[44] Sulistyaningsih E, Loeki EA, Loscher T, Berens-Riha N (2010). Diagnostic difficulties with Plasmodium knowlesi infection in humans. Emerg Infect Dis..

[45] Elyazar IR, Hay SI, Baird JK (2011). Malaria distribution, prevalence, drug resistance and control in Indonesia. Adv Parasitol..

[46] Baird JK (2009). Resistance to therapies for infection by Plasmodium vivax. Clin Microbiol Rev..

[47] Abdussalam R, Krimadi RNI, Siregar R, Lestari ED, Salimo H (2016). Profil infeksi plasmodium, anemia dan status nutrisi pada malaria anak di RSUD Scholoo Keyen. Kabupaten Sorong Selatan. Sari Pediatri..

[48] Subekti N, Kawulur Paiticen M, Sirait SHK EIJJ, Mohammed S (2018). Types of plasmodium and the effect of environmental factor against malaria in Manokwari, West Papua. JPII..

[49] Anabire NG, Aryee PA, Helegbe GK (2018). Hematological abnormalities in patients with malaria and typhoid in Tamale Metropolis of Ghana. BMC Res Notes..

[50] Durrani SH, Sayyar M, Lal A, Aslam R (2015). Incidentally diagnosed bicytopenia showing a wide spectrum of pathologies on bone marrow morphology. KJMS..

[51] Sweta Barik S, Chandoke RK, Verma AK (2014). A prospective clinico-hematological study in 100 cases of pancytopenia in capital city of India. J Appl Hematol..

[52] Kenangalem E, Karyana M, Burdarm L, Yeung S, Simpson JA, Tjitra E (2016). *Plasmodium vivax* infection: a major determinant of severe anaemia in infancy. Malar J..

[53] Barber BE, William T, Jikal M, Jilip J, Dhararaj P, Menon J (2011). *Plasmodium knowlesi* malaria in Children. Emerg Infect Dis..

[54] Price RN, Tjitra E, Guerra CA, Yeung S, White NJ, Anstey NM (2007). Vivax malaria: neglected and not benign. Am J Trop Med Hyg..

[55] Menendez C, Fleming AF, Alonso PL (2000). Malaria-related anaemia. Parasitol Today..

[56] Ugwu EO, Dim CC, Uzochukwu BS, Iloghalu EI, Ugwu AO (2014). Malaria and anaemia in pregnancy: a cross-sectional study of pregnant women in rural communities of Southeastern Nigeria. Int Health..

[57] Price RN, Simpson JA, Nosten F, Luxemburger C, Hkirjaroen L, ter Kuile F (2001). Factors contributing to anemia after uncomplicated falciparum malaria. Am J Trop Med Hyg..

[58] Quintero JP, Siqueira AM, Tobón A, Blair S, Moreno A, Arévalo-Herrera M (2011). Malaria-related anaemia: a Latin American perspective. Mem Inst Oswaldo Cruz..

[59] Kitua AY, Smith TA, Alonso PL, Urassa H, Masanja H, Kimario J (1997). The role of low level Plasmodium falciparum parasitaemia in anaemia among infants living in an area of intense and perennial transmission. Trop Med Int Health..

[60] Perrin LH, Mackey LJ, Miescher PA (1982). The hematology of malaria in man. Semin Hematol..

[61] Clark IA, Chaudhri G (1988). Tumour necrosis factor may contribute to the anaemia of malaria by causing dyserythropoiesis and erythrophagocytosis. Br J Haematol..

[62] Dondorp AM, Angus BJ, Chotivanich K, Silamut K, Ruangveerayuth R, Hardeman MR (1999). Red blood cell deformability as a predictor of anemia in severe falciparum malaria. Am J Trop Med Hyg..

[63] George LO, Ewelike-Ezeani CS (2011). Haematological changes in children with malaria infection in Nigeria. Journal of Medicine and Medical Science..

[64] Fattahi BA, Hashemi A, Abolhasanizadeh S (2015). Comparison of hematological aspects among children with malaria and healthy children. Iran J Ped Hematol Oncol..

[65] Ladhani S, Lowe B, Cole AO, Kowuondo K, Newton CR (2002). Changes in white blood cells and platelets in children with falciparum malaria: relationship to disease outcome. Br J Haematol..

[66] Beale PJ, Cormack JD, Oldrey TB (1972). Thrombocytopenia in malaria with immunoglobulin (IgM) changes. Br Med J..

[67] Skudowitz RB, Katz J, Lurie A, Levin J, Metz J (1973). Mechanisms of thrombocytopenia in malignant tertian malaria. BMJ..

[68] Essien EM (1989). The circulating platelet in acute malaria infection. Br J Haematol..

[69] Pain A, Ferguson DJ, Kai O, Urban BC, Lowe B, Marsh K (2001). Platelet-mediated clumping of Plasmodium falciparum-infected erythrocytes is a common adhesive phenotype and is associated with severe malaria. Proc Natl Acad Sci U S A..

[70] Brouwers J, Noviyanti R, Fijnheer R, de Groot PG, Trianty L, Mudaliana S (2013). Platelet activation determines angiopoietin-1 and VEGF levels in malaria: implications for their use as biomarkers. PLoS ONE..

[71] Gérardin P, Rogier C, Ka AS, Jouvencel P, Brousse V, Imbert P (2002). Prognostic value of thrombocytopenia in African children with falciparum malaria. Am J Trop Med Hyg..

[72] Lathia TB, Joshi R (2004). Can hematological parameters discriminate malaria from nonmalarious acute febrile illness in the tropics?. Indian J Med Sci..

[73] Kotepui M, Phunphuech B, Phiwklam N, Chupeerach C, Duangmano S (2014). Effect of malarial infection on haematological parameters in population near Thailand-Myanmar border. Malar J..

[74] Goyal JP, Makwana AM (2014). Comparison of clinical profile between P. vivax and P. falciparum malaria in children: a tertiary care centre perspective from India. Malar Res Treat..

[75] Nandwani S, Pande A, Saluja M (2014). Clinical profile of severe malaria: study from a tertiary care center in north India. J Parasit Dis..

[76] Sajjanar AB, Dinesh US, Kanbur D, Mane V, Athanikar V (2013). Hematological parameters in Plasmodium Vivax and Falciparum Malaria-a study at tertiary care centre in North Karnataka. NJLM..

[77] George P, Alexander L (2010). A study on the clinical profile of complicated Plasmodium vivax mono–infections. Asian Pac J Trop Med..

[78] Rodríguez-Morales AJ, Sánchez E, Vargas M, Piccolo C, Colina R, Arria M (2006). Anemia and thrombocytopenia in children with Plasmodium vivax malaria. J Trop Pediatr..

[79] Srivastava S, Ahmad S, Shirazi N, Verma SK, Puri P (2011). Retrospective analysis of vivax malaria patients presenting to tertiary referral centre of Uttarakhand. Acta Trop..

[80] Rogerson SJ, Carter R (2008). Severe vivax malaria: newly recognised or rediscovered. PLoS Med..

[81] Genton B, Mueller I (2010). Vivax malaria: more severe and more resistant. Therapy..

[82] Price RN, Douglas NM, Anstey NM (2009). New developments in Plasmodium vivax malaria: severe disease and the rise of chloroquine resistance. Curr Opin Infect Dis..

[83] World Health Organisation (2014). Severe malaria. Trop Med Int Health..

[84] Saravu K, Rishikesh K, Kamath A, Shastry AB (2014). Severity in Plasmodium vivax malaria claiming global vigilance and exploration-a tertiary care centre-based cohort study. Malar J..

[85] Gupta P, Sharma R, Chandra J, Kumar V, Singh R, Pande V (2016). Clinical manifestations and molecular mechanisms in the changing paradigm of vivax malaria in India. Infect Genet Evol..

[86] Wickramasinghe SN, Abdalla SH (2000). Blood and bone marrow changes in malaria. Best Pract Res Clin Haematol..

[87] Greenwood BM, Armstrong JR (1991). Comparison of two simple methods for determining malaria parasite density. Trans R Soc Trop Med Hyg..

[88] Nwanjo HU, Opara AU (2005). Effects of falciparum malaria infection on some haematological indices and renal functions. J Med Lab Sci..

[89] Kueh YK, Yeo KL (1982). Haematological alterations in acute malaria. Scand J Haematol..

[90] Lisse IM, Aaby P, Whittle H, Knudsen K (1994). A community study of T lymphocyte subsets and malaria parasitaemia. Trans R Soc Trop Med Hyg..

[91] Reyburn H, Mbatia R, Drakeley C, Carneiro I, Mwakasungula E, Mwerinde O (2004). Overdiagnosis of malaria in patients with severe febrile illness in Tanzania: a prospective study. BMJ..

[92] Crawley J, Chu C, Mtove G, Nosten F (2010). Malaria in children. Lancet..

[93] Baird JK, Krisin Barcus MJ, Elyazar IRF, Bangs MJ, Maguire JD (2003). Onset of clinical immunity to Plasmodium falciparum among Javanese migrants to Indonesian Papua. Ann Trop Med Parasitol..

